# Discovering Social Events through Online Attention

**DOI:** 10.1371/journal.pone.0102001

**Published:** 2014-07-30

**Authors:** Dror Y. Kenett, Fred Morstatter, H. Eugene Stanley, Huan Liu

**Affiliations:** 1 Center for Polymer Studies and Department of Physics, Boston University, Boston, Massachusetts, United States of America; 2 School of Computing, Informatics, and Decision Systems Engineering, Arizona State University, Tempe, Arizona, United States of America; University of Maribor, Slovenia

## Abstract

Twitter is a major social media platform in which users send and read messages (“tweets”) of up to 140 characters. In recent years this communication medium has been used by those affected by crises to organize demonstrations or find relief. Because traffic on this media platform is extremely heavy, with hundreds of millions of tweets sent every day, it is difficult to differentiate between times of turmoil and times of typical discussion. In this work we present a new approach to addressing this problem. We first assess several possible “thermostats” of activity on social media for their effectiveness in finding important time periods. We compare methods commonly found in the literature with a method from economics. By combining methods from computational social science with methods from economics, we introduce an approach that can effectively locate crisis events in the mountains of data generated on Twitter. We demonstrate the strength of this method by using it to locate the social events relating to the Occupy Wall Street movement protests at the end of 2011.

## Introduction

Over the past several years various Internet social media platforms have enabled people to communicate, locate resources, and disseminate information during times of turmoil, e.g., natural disasters, health epidemics, or social unrest. Twitter, one major social media platform, has emerged as a leading social media outlet. With 200 million users sharing 140-character text messages (“tweets”) over 400 million times each day [1], Twitter's popularity and influence on world events have made it a hot topic for social media research [2]. Research on Twitter began in 2010 when researchers saw its potential for rapid communication and information diffusion. The field of computational social science has been rapidly expanding in response to the influence of Twitter and other online social platforms [3], [4], and new insights into social structure and social dynamics are emerging [5]–[15]. Twitter has also been a focus in studies of humanitarian assistance/disaster relief (HA/DR) efforts [16]–[18] and in the tracking of disease epidemics [19]. Because Twitter enables the real-time propagation of information to large groups of users, it is an ideal environment for the dissemination of breaking news from news gatherers and from on-site locations where events are taking place.

Twitter has several features of interest to the research community. Twitter's “retweet” feature, which allows users to push content through the network by forwarding it to their followers, has elicited much research on how information propagates in social media [20], [21], how retweets facilitate online conversation [22], and how retweets factor in times of crisis [23]. Twitter uses a special text feature (a “hashtag”) in which transmitted words are prefixed with a “#” sign. Every hashtag has a page showing the history of all the tweets containing that hashtag in the text, and this creates a community of users discussing that particular hashtag [24]. This encourages users interested in the topic to use the associated hashtag in their tweets to increase the audience of their tweet, and the study of this tagging behavior in Twitter has become an extremely active area of research [25]–[27]. In addition to text, users can also annotate their tweet with their current location, adding what is called a “geotag.” Only about one percent of all tweets are geotagged, yet they still provide background information about an event. Recent work has focused on combining location with textual content to detect topics more relevant to specific regions [28]–[30]. Because geotags are so sparse, recent work has also focused on associating non-geotagged tweets with a location to better understand the context of the tweet [31], [32].

Social media platforms now strongly factor in the spreading of ideas and the organization of social movements. Over the past few years, social media has played a key role in such significant events as the Arab Spring uprisings and the violent demonstrations organized in London. Twitter is popular with users seeking to spread information about a cause. Because each message can be no longer than 140 characters, communication spreading information concerning protest gatherings, earthquake relief, or the location of aid stations is extremely rapid [33], [34]. Participants in the Arab Spring used Twitter to quickly coordinate protests [35], [36]. Occupy Wall Street, a movement protesting the wealth disparity in the United States, was largely organized on Twitter under the hashtag “#OccupyWallStreet.” As the movement spread and authorities began to retaliate, protesters used Twitter to report abuses by police, thus bringing more attention to their cause. Social media became so central during the Arab Spring protests that the regimes in such countries as Egypt and Syria cut the protesters' access to the Internet. During Hurricane Sandy, authorities used Twitter to spread news of power outages and the locations of resources for those affected by the storm.

Because Twitter provides rapid communication and information diffusion, millions of people use it to keep up with current events and create their own discussion threads. Because activity on the Twitter site is huge, it is difficult to differentiate periods of focused discussion from periods of casual chatter. How do we identify the key periods of discussion? How do we filter out the noise and locate the main issues of discussion people are discussing at any given time?

We will first attempt to locate the periods where tweets reflect actual events on the ground. To harness the abundance of data produced by Twitter, we need a highly-scalable method to find key time periods of big events in social media. We focus on the Twitter activity surrounding Occupy Wall Street–the vast Twitter discussion of that event worldwide–and compare several methods of quantifying social communication.

## Occupy Wall Street Movement

The Occupy Wall Street movement began on 17 September 2011 in New York City. The movement was largely promoted on social media, and many hashtags were used to discuss the event. The chief driving force behind this movement was the growing wealth disparity between rich and poor in the United States [37]. As the movement gained attention, other Occupy movements emerged in cities across the US. As citizens in other countries identified with the core concerns of the movement, similar actitivies spread across the globe. By 15 October 2011, 951 similar protests had occurred in 82 countries [38]. As the movement continued to grow it was officially endorsed by a number of city governments and labor unions [39].

In this study we collected tweet data from 14 September 2011 through 3 April 2012 using the parameters shown in Table 1 and encompassing 15,736,835 tweets with 402,758 unique hashtags and 6,967,392 retweets. We used Twitter's free, publicly-available data source, the Streaming API (see https://dev.twitter.com/docs/streaming-apis) to collect the data, in which three parameters are supported: keywords (which can be supplied in the form of words, phrases, or hashtags), locations (supplied as a geographic bounding box), and users. Every parameter is treated as an “OR” condition. That is, a tweet will be returned from the Streaming API if it contains at least one of the keywords, if it is produced from within the bounding box using a “geotag”, or if it is authored by one of the users specified in the parameters. When a user geotags their tweet, their location is provided as part of the metadata using the GPS sensor on their device (for more information see http://support.twitter.com/articles/78525-faqs-about-the-tweet-location-feature). All parameters supplied to (and tweets returned by) the Streaming API were managed using TweetTracker [40].

**Table 1:** Parameters supplied to the Streaming API for each of the data sources.

Data Set	Keywords	Geoboxes	User Timelines
Occupy Wall Street	#occupywallstreet, #ows, #occupyboston, #p2, #occupywallst, #occupy, #tcot, #occupytogether, #teaparty, #99percent, #nypd, #takewallstreet, #occupydc, #occupyla, #usdor, #occupysf, #solidarity, #15o, #anonymous, #citizenradio, #gop, #sep17, #occupychicago, #occupyphoenix, #occupyoakland	None	None

Coordinates below the boundary box indicate the Southwest and Northeast corner, respectively. No users were tracked during the course of data collection.

**Figure 1:**
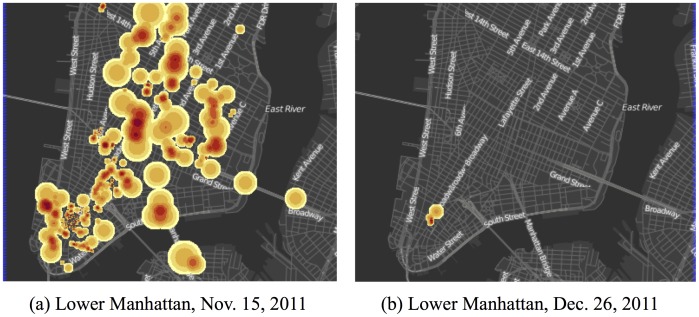
Heatmap of geotagged Twitter activity. Twitter activity related to the Occupy Wall-Street (OWS) Movement, collected for hashtags, or topics, used by protests or members of the movement. The “redder” areas indicate regions with more tweets. Here we see two extremes of geotagging behavior. Panel (a) shows the tweets for 15 November 2011, when the New York Police Department attempted to remove protesters from Zuccotti Park. Panel (b) shows the tweets for 26 December 2011, when protesting had dwindled. In between these two extremes of activity, is a more general pattern of discussion centered around the protests in Zuccotti Park.

Many of the tweets collected were geotagged, with a large number of the geotagged tweets coming from New York City. Figure 1 shows a heatmap of the tweets produced on different days and we can see the extreme cases of geotagged tweets. Figure 1(a) shows the tweets for 15 November 2011, when the New York Police Department attempted to remove protesters from Zuccotti Park. Figure 1(b) shows the tweets for 26 December 2011, when protesting had dwindled. In between these two extremes of activity, is a more general pattern of discussion centered around the protests in Zuccotti Park.

## Measures of Social Attention

The Herfindahl-Hirschman index (also known as the Herfindahl index, or HHI) is a measure of the size of firms in relation to an industry and indicates the degree of competition among them. Named after economists Orris C. Herfindahl and Albert O. Hirschman, it is an economic concept widely applied in competition law, antitrust law, and technology management. The measure is also used by the United States Department of Justice when evaluating mergers (see http://www.justice.gov/atr/public/guidelines/hhi.html). The result is proportional to the average market share, weighted by market share. As such, it can range from 0 to 1, moving from a huge number of very small firms (with a value reaching zero) to a single monopolistic producer (with a value reaching 1). Increases in HHI generally indicate a decrease in competition and an increase of market power, whereas decreases indicate the opposite.

We use a normalized HHI [42], *H* ^*^, which is defined as 
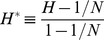
(1)where 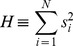
(2)
*N* is the number of hashtags, and *s* is the percentage of the aggregate measure (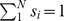).

We utilize the HHI as a “thermostat” of social attention. Each hashtag represents a “firm” and the number of users tweeting this hashtag relative to the total number of users in a given time period represents the hashtag's “market cap.” This enables us to examine the HHI value of different hashtags for a given time period. High HHI values indicate a strong focus on a specific topic, and low HHI values indicate a diffused focus among a wide variety of topics.

**Figure 2:**
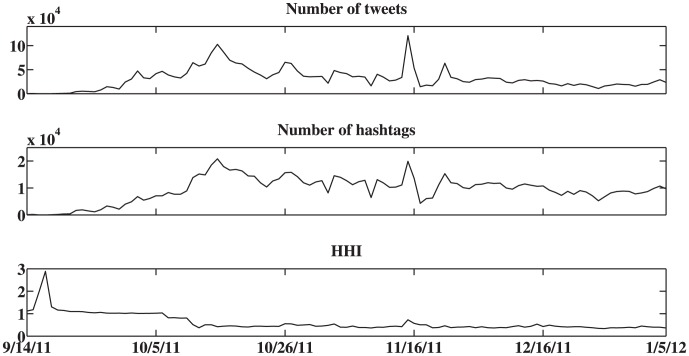
Time evolution of the number of tweets (top), number of hashtags (middle), and Herfindahl-Hirsch Index (HHI) parameter (bottom) for the OWS dataset, on a daily time horizon. The HHI calculates how diverse the discussion is on Twitter, by calculating how many messages are associated with a given hashtag, and ranges from a value of 0, for highly diverse discussion, to 1, when all messages are focused on only one hashtag.

**Figure 3:**
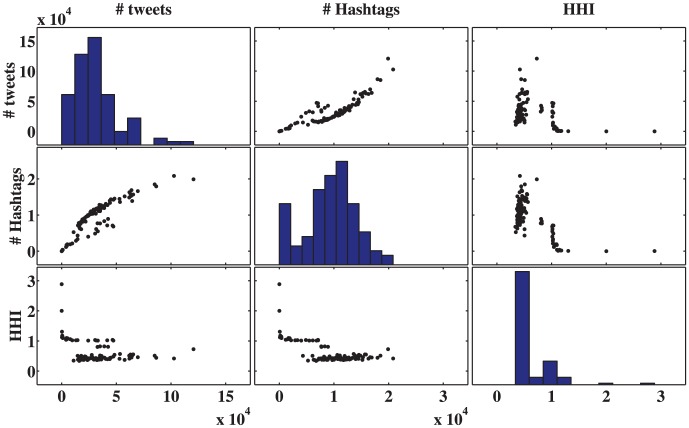
Comparison of the HHI to its underlying parameters: the number of tweets, and number of hashtags. Here, the diagonal figures represent the histogram of values for each of these three parameters, whereas the off diagonal panels represent a comparison of the values of two different parameters. It is clear by studying these figures that the HHI is not merely a function of either the number of tweets or number of users.

We use HHI analysis to study the OWS dataset and calculate the HHI value for a time horizon of a single day, using the number of users and hashtags. One concern of the HHI is that it is dependent on the number of tweets produced in a given time interval. Figure 2 shows the time evolution of the HHI. Figure 3 compares the HHI with its underlying parameters: the number of users and the number of hashtags. Here the diagonal figures represent the histogram of values for each of these three parameters, whereas the off-diagonal panels represent a comparison of the values of two different parameters. Studying this figure, it is clear that the HHI is not merely a function of either of these two parameters.

Another attention-based measure of social attention is the entropy [43] of the hashtags over a given time period. We here consider the hashtag probability to be the number of times the hashtag is used over the number of times all hashtags are used in a given time interval. The hashtag entropy is calculated by first assigning the probability of a given hashtag, *p_i_*, using the fraction of users who tweeted this hashtag in the given time horizon, summing over all hashtags such that: 
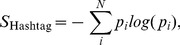
(3)where *N* is the number of hashtags in the given time horizon. In evaluating the effectiveness of our HHI-based approach, we compare its performance as a classifier of the ground truth relative to that of the other three models.

## Indicators of Activity in Social Media

To search for periods of focused discussion, we locate time periods with a large number of tweets or time periods with a large number of unique hashtags and test whether these two simple measures can enable us to identify the focused discussion periods in the dataset. We quantitatively test the two simple measures by performing a receiver operating characteristic (ROC) curve analysis. The ROC curve plots the fraction of true positives out of the positives and the fraction of false positives out of the negatives for a binary classifier system. ROC curve analysis is a standard method in signal detection theory as well as in psychology, medicine, and biometrics [41]. One key measure from the ROC curve is the area-under-curve (AUC) score, the measure of the area under the ROC curve. The ROC AUC varies from 0.50 (totally random classification) to 1.0 (perfect classification).

**Figure 4:**
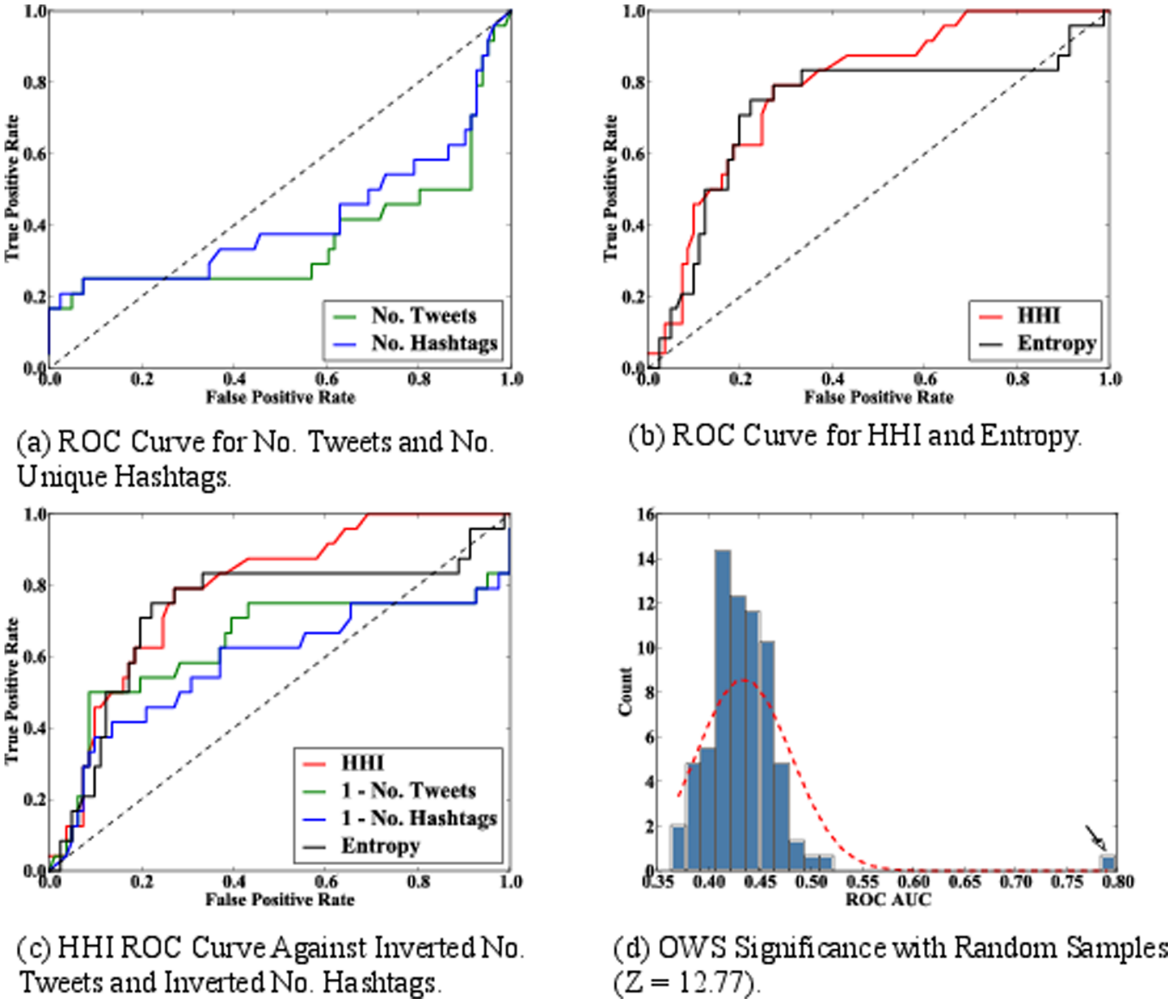
HHI ROC analysis. (a) ROC curve of number tweets and number unique hashtags as classifiers for finding significant dates in the dataset. Number of tweets AUC = 0.42 and number of unique hashtags AUC = 0.36. (b) ROC curve of the HHI and Entropy classifiers. HHI AUC = 0.79, entropy AUC = 0.72. The focus-based classifiers provide the best classification when compared with the other methods, with the HHI being the best predictor. (c) ROC curve of the four classifiers - one minus number of tweets, one minus number of hashtags, and hashtag entropy - and their performance in identifying the ground truth. This is done as a below-random (<0.50) AUC means that the class labels should be inverted. (d) Distribution of the HHI AUC values for prediction of the ground truth for many random samples of the OWS dataset. The arrow in this figure represents the measure of the unshuffled data.

We vary the measurement threshold to identify important days, and compare the results with the ground truth. The true positive rate is defined as the fraction of the actual significant days, as listed by the ground truth, that are also identified by the measure. The false positive rate is the fraction of days that are not identified in the ground truth, but are identified as significant by the measure. Each point in the ROC curve corresponds to one selection threshold. A random classifier yields a diagonal line (AUC = 0.50) from the bottom-left to the top-right corner. The greater the curve's distance above the diagonal line, the stronger the model's predictive power. To obtain ground truth, we extract dates from the Wikipedia timeline of the OWS protests (see http://en.wikipedia.org/wiki/Timeline_of_Occupy_Wall_Street). Next, by varying the threshold that indicates “important” days, we find the ROC curve, shown in Figure 4(a). The ROC AUC of the top hashtags is 0.36 and the ROC AUC of the top tweets is 0.42, both scoring worse than a perfectly random classifier.

Although we can mitigate the poor results obtained in the experiment by inverting the class labels–giving the inverted hashtag and tweet indicators ROC AUCs of 0.64 and 0.58, respectively–this approach has intuitive problems. Predicting periods with few unique hashtags and few tweets is not relevant to the problem of finding periods of intense discussion. Therefore, there is a need for a measure of social attention that focuses not only on the number of tweets or unique hashtags, but also on their “attention”–the degree to which users congregate around them.

## Social Attention as a Detector of Real-World Events

We next use the HHI as a thermostat for social focus during times of crisis. Alternate approaches would be to use the number of tweets, the number of unique hashtags produced in a given day, or the entropy of the hashtags used in the time period.

Figure 4(b) and Figure 4(c) shows the results of performing all four indicators on the OWS dataset, with HHI and entropy attaining ROC values of 0.79 and 0.72, respectively. The attention-based indicators provide the best classification when compared with the other methods, with the HHI being the best predictor.

To confirm that the classification accuracy of the HHI comes from the hashtag selection made by the users and is not merely an artifact of the volume of tweets, we randomly shuffle the tweets based on the time they were produced. If the effectiveness of the HHI is due to the volume of tweets, then there should be no significant difference between the initial AUC and those from the datasets with the randomly shuffled timestamps.

To this end, we create a unique set, *T*, of all the timestamps from tweets in the dataset. For each tweet we then randomly choose a timestamp from *T* and assign it to the tweet, without replacement. Using this shuffled dataset we calculate the ROC AUC score. We repeat this process 100 times to determine the distribution of the shuffled tweets. Finally, we compare the AUC score of the original data with the shuffled data to see if it differs significantly (*μ*±3σ) from the center of the random shuffles. Figure 4(d) shows the distribution of ROC AUC scores of the randomly shuffled data. The Z–score of the original data, calculated as 
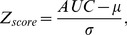
(4)is +12.77, significantly outside of the control bounds.

## Summary

In this work we investigate the problem of finding real-world events quickly as they unfold in large, noisy social media data. We seek to find a measure of attention in social media. The naive choice for this aim is to investigate the number of tweets and number of unique hashtags, and we find that this approach is unsatisfactory. One possible explanation for the poor performance of these measures could be that extraneous conversation on Twitter leads to spikes in activity not relevant to the event. We investigate two additional methods, HHI and entropy, and find that they are successful detectors of these periods of intense discussion. HHI, a measure borrowed from the economics literature adapted for use in social media, yields the best results for identifying the times of intense discussion.

Our results indicate that significant social events cause the discussion on Twitter to move from many subjects to a few, as demonstrated through the Herfindahl index. In terms of classical information theory, this can be conversely related to a measure of entropy of the discussion topics, where our results show that significant events are related to drops in the entropy (or high HHI). Entropy has been used in the past to study traditional media and online media [44]–[46]. Our results show that while the two measures are closely related, the HHI outperforms entropy as a detector of significant events. This work presents a first use of the HHI to study social attention on Twitter.

Although discussions in Twitter and in digital social media in general are extremely heterogeneous, when a significant event occurs discussions converge to the event and become extremely homogeneous. The point at which this switching occurs indicates the magnitude of the event. Because of this, the proposed Herfindahl index provides a means of detecting significant events, and provides a simple measure to filter significant events and centers of attention in the social online media. This simple yet sophisticated measure can provide important insights to people of different background and needs, such as scientists, social-media based marketing professionals, policy and decision makers, and a multitude of relief agency workers.
